# Psychological resilience in Olympic combat sports

**DOI:** 10.3389/fpsyg.2025.1605765

**Published:** 2025-06-13

**Authors:** Radu Predoiu, Maurizio Bertollo, Andrzej Piotrowski, Rareș Stănescu, Faten Hamdi, Gabriela Szabo, Germina Cosma

**Affiliations:** ^1^Department of Teachers Training, Faculty of Physical Education and Sport, National University of Physical Education and Sports, Bucharest, Romania; ^2^Department of Medicine and Aging Sciences, School of Medicine and Health Sciences, University “G. d’Annunzio” of Chieti-Pescara, Chieti, Italy; ^3^Institute of Psychology, University of Gdańsk, Gdańsk, Poland; ^4^Department of Sports and Motor Performance, Faculty of Physical Education and Sport, National University of Physical Education and Sports, Bucharest, Romania; ^5^High Institute of Sport and Physical Education, University of Jendouba, El Kef, Tunisia; ^6^Advanced Medical and Pharmaceutical Research Center, "George Emil Palade" University of Medicine, Pharmacy, Science and Technology, Târgu Mureș, Romania; ^7^Faculty of Physical Education and Sport, University of Craiova, Craiova, Romania; ^8^InnovaSport Craiova Interdisciplinary Laboratory, INCESA, Craiova, Romania

**Keywords:** psychological resilience, combat sports, sports performance, martial arts, caliber

## Abstract

**Introduction:**

The purpose of the study was to examine psychological resilience in Olympic combat sports, comparing gender, sports performance level and discipline type. Moreover, we verified whether resilience predicts sports performance.

**Materials and methods:**

Eighty-four athletes were involved in the study. Psychological resilience was assessed with the Romanian adaptation of the Brief Resilience Scale.

**Results:**

Using the Goodman and Kruskal tau association test a significant link was found between athletes’ gender and the scoring on psychological resilience. Also, analysis of variance and Tukey *post-hoc* test highlighted significant differences between athletes’ level (i.e., international, national, and regional/local athletes) (*p* = 0.02, respectively *p* < 0.01). Data analysis showed no significant differences in resilience (*p* = 0.182) between the Olympic combat sports (disciplines) investigated (boxing, karate, fencing and taekwondo). In addition, a binomial logistic regression was performed, predicting athletes’ likelihood to obtain higher sports performances based on psychological resilience.

**Conclusion:**

A slightly above average level of psychological resilience (generally) is linked with an increased likelihood of international and/or national performances in Olympic combat sports. On the other hand, athletes with lower caliber obtained the highest scores for resilience. In addition, male athletes obtained higher scores for resilience than female athletes. The study offers a valuable window into understanding psychological resilience in combat sports.

## 1 Introduction

The term resilience comes from the Latin word *resilire* which means to bounce back or to return in the original form (Pânișoară, 2024), referring to an organism’s ability to adapt, to cope with pressure when faced with stressful situations (Kent et al., 2013). The psychological attribute of resilience appeared in the newspapers already in 1893 when the Independent of New York published “The resilience and the elasticity of spirit which I had even 10 years ago” (Oxford English Dictionary, 2025). Resilience is seen as a personal adaptive quality, which helps a person to withstand distress (Ahern et al., 2008), it is the individual’s ability to be flexible in the face of behaviors, thoughts, feelings when faced with prolonged pressure, ultimately becoming stronger and wiser (Pemberton, 2015). Resilience is thus not only the ability to bounce back after failures or challenges, but also the ability to be stronger and achieve superior results in the work performed (Fletcher and Sarkar, 2013). Resilience protects the individual from psycho-emotional disorders (Yang et al., 2020), facilitates growth and development (Shi et al., 2019).

Most definitions for resilience include the following concepts: positive adaptation, rebound/bouncing back and maintenance of wellbeing (in face of adversity) (Bryan et al., 2019). Masten and Wright (2010) specify that resilience should be defined as a dynamic process and an interaction of the individual with his/her changing environment (not only as an individual characteristic). Resilience is influenced by the neural and psychological organization of the person, by the interaction between the life context and the developing organism (Curtis and Cicchetti, 2007). Bryan et al. (2023) mention, also, that “future resilience research requires a shift in perspective away from resilience as a trait […] and toward resilience as a process influenced by a multitude of asset and risk factors.”

In the sporting context, resilience improves personal skills and protects injured athletes from the negative effects of various stressors (Zurita-Ortega et al., 2018). The aforementioned researchers found a positive link between soccer players’ resilience and their ability to successfully adapt to injuries. A more resilient person will return more quickly to normal performance after a setback, i.e., injury. The characteristics of a resilient person include participation in physical activities, optimistic view of the future, positive attitude toward challenges, predominant positive emotions, developing a sense of belonging to a group, has self-confidence and mobilization in the face of challenges to overcome them, view problems from multiple perspectives, actively engagement in social networks of trust (Sutton, 2019). Indeed, athletes are able to attribute a positive meaning to complex events through resilience, cope with negative emotions and adapt to external stressors (Xu et al., 2021). Resilient persons activate divergent thinking (Predoiu and Predoiu, 2024), an aspect that makes it possible to understand problems from multiple points of view/perspectives, to activate different explanations and find possible solutions (Sánchez et al., 2015).

Only recently practitioners and researchers have started to examine psychological resilience as a construct, within the sport domain (Galli and Gonzalez, 2015), reviewing also the stressors and protective factors (Sarkar and Fletcher, 2014). Galli and Vealey (2008), for example, compared psychological resilience between former and current high-level athletes, while Mummery et al. (2004) investigated resilience level in swimmers. Blanco-García et al. (2021) analyzed how resilience varies across different sports, genders, ages, and competitive levels in a sample of 1,047 athletes from five sports: handball, basketball, volleyball, athletics, and judo. The study employed the Brief Resilience Scale (BRS) to assess resilience levels, and the findings suggest important variations based on gender and age, but not on the sport type or competitive level.

More mentally resilient athletes cope better with the pain experienced, are more motivated to participate in recovery sessions (e.g., after an injury), manage to remain positive and emotionally more stable and overcome critical periods more quickly (Berceanu, 2024; Kaiseler et al., 2009). Psychological resilience influences athletes’ recovery time (and beyond) from concussions suffered on the field. Conversely, low resilience scores predict a more difficult recovery (Ernst et al., 2022), and are related to higher scores for depressive symptoms and anxiety during recovery (Bunt et al., 2021). Resilience has also been associated with better adaptation in individuals who have suffered brain injury (Neils-Strunjas et al., 2017).

According to the sports resilience meta-model (Gupta and McCarthy, 2022), disruptions will be smaller or larger in athletes, depending on how the biopsychosocial protective filter operates. Fletcher and Sarkar (2012) discuss positive thinking, confidence, motivation, focus, and perceived social support as essential factors in developing resilience, supporting athletes in the face of challenges. Under pressure, athletes may experience unsportsmanlike behaviors (Vansteenkiste et al., 2010), reduced self-esteem (Gagné et al., 2003), being, also, more prone to burnout (Tabei et al., 2012). In these conditions, athletes need to adapt to adversities on their “*road*” to great performance (Gould et al., 2002).

Most of the previous studies analysed resilience through the lens of a cognitive-behavioral approach, menawhile Hill et al. (2018) proposed a dynamics systems approach to understanding resilience in athletes. The authors argued that resilience should be seen as a process that evolves over time through the interaction of various factors, including psychological and physiological responses to adversity. They highlighted the importance of viewing resilience as dynamic rather than static phenomenon, emphasizing the role of fluctuations in performance and recovery.

Specifically, in martial arts and combat sports, resilience is a crucial trait that involves both mental and physical dimensions, shaping a practitioner’s capacity to overcome challenges, stay focused, and constantly improve. It is vital not only for mastering physical techniques but also for developing the mental toughness needed to excel in the discipline (Fletcher and Sarkar, 2012). In judo, Jo (2016) highlighted resilience’s potential to decrease exhaustion and distress. It seems that practice time and schooling level influence martial artists’ level of resilience (da Gama et al., 2018). Also, Moore et al. (2021) underlined the positive effects of martial arts-based interventions in developing resilience.

A key component of martial arts is physical resilience, as training often includes intense workouts, sparring, and repetitive drills that push the body to its limits. Practitioners must endure pain, fatigue, and occasional injuries, which require physical resilience to overcome. This is developed through regular practice, conditioning, and learning how to recover from physical strain. Martial artists focus on enhancing their strength, flexibility, endurance, and overall physical fitness to withstand these challenges (Fletcher and Sarkar, 2012). Equally important, if not more so, is mental resilience – the ability to cope with stress, frustration, and failure. Martial arts training is often designed to test a practitioner’s mental endurance. Practitioners encounter various challenges, such as mastering difficult techniques, facing defeat in sparring or competition, and overcoming self-doubt. Mental resilience enables them to push through these obstacles and stay focused on their goals, even in tough situations (Galli and Vealey, 2008). A key part of mental resilience is adopting a mindset of continuous improvement. Martial artists are encouraged to view setbacks as learning opportunities rather than failures. This growth mindset nurtures perseverance, driving individuals to persist in the face of challenges and remain determined to improve. Overcoming psychological barriers, such as the fear of failure or injury, is essential in building resilience. Not least, psychological resilience was explored in connection with the Big Five personality factors (Rawat et al., 2023) – athletes from Olympic combat sports (boxing, karate, fencing, taekwondo) being included in the sample.

### 1.1 The current study

The main *aim* of our study was to investigate psychological resilience in Olympic combat sports, according to gender, type of sport and sports performance. A secondary aim of our study was to verify if resilience predicts performance in combat sport athletes.

*Objectives*

- Understanding the resilience level in athletes practicing striking combat sports (e.g., boxing, taekwondo);
- Identifying the differences in psychological resilience between combat sport athletes, taking into consideration their performance;
- Establishing the differences between sports and exploring gender-related associations, in the context of psychological resilience.

*Hypotheses*

*H_1_*: Investigation of psychological resilience shows sport-related differences, in Olympic combat sports.

*H_2_*: There are gender-related associations in terms of resilience, in combat sport athletes.

*H_3_*: Investigation of psychological resilience reveals significant differences between martial artists, according to sports performances level.

*H_4_*: Resilience predicts sports performance in Olympic combat sports.

## 2 Materials and methods

### 2.1 Participants

Eighty-four athletes practicing Olympic combat sports, affiliated to different sports clubs in Romania, participated in the study, 61 male and 23 female, aged 18–27 years (*M*_age_ = 20.6 years, *SD* = 4.26). Athletes were practicing Olympic combat sports, being systematically involved in training and competitions. Inclusion criteria were: ≥18 years old and minimum 2 years of competitive experience (*M*_competitive experience_ = 4.95 years, *SD* = 2.16). Also, the presence of the sport discipline in at least one of the last two Olympic Games: Tokyo, 2020, respectively Paris, 2024, represented an inclusion criterion in the research.

Considering sports experience and level: 26 combat sport athletes (30.95%) of which 9 female athletes obtained international performances (top ranks at World and/or European competitions); 31 athletes (36.9%) of which 7 female athletes registered National sports results (top ranks at national competitions), and 27 athletes (32.14%) of which 7 female athletes obtained Regional/ Local level results (at county level). Therefore, our athletes can be categorized as part of Tier 3, 4, and 5 (McKay et al., 2022). The convenience and the snowball sampling technique (Tashakkori and Teddlie, 2003) were used to identify the senior combat sport athletes.

The distribution of the participants among sport can be seen in Table 1.

**Table 1:** Distribution of study participants.

Olympic combat sports	*N*	Male athletes	Female athletes
Boxing	24 (28.57%)	21 (34.4%)	3 (13.04%)
Karate	26 (30.95%)	18 (29.5%)	8 (34.78%)
Taekwondo	16 (19.05%)	13 (21.31%)	3 (13,04%)
Fencing	18 (21.43%)	9 (14.75%)	9 (39.13%)
Total	84 (*M*_age_ = 20.6 yeas)	61 (*M*_age_ = 20.8 yeas)	23 (*M*_age_ = 20.2 yeas)

### 2.2 Measures and procedure

Psychological resilience was assessed with The Romanian adaptation of the Brief Resilience Scale (BRS). The BRS (in its Romanian adaptation) “revealed adequate fit-indexes,” and “suitable values were also obtained for reliability and convergent validity” (Alexe et al., 2022). BRS consists of six items (three direct scoring and three reverse scoring items), e.g., “I usually come through difficult times with little trouble” (direct scoring item), “I tend to take a long time to get over set-backs in my life” (reverse scoring item) (Smith et al., 2008). Answer options: 1 “Strongly Disagree,” 2 “Disagree,” 3 “Neutral,” 4 “Agree,” 5 “Strongly Agree.” The sum range from 6 to 30, and for the final score the sum is divided by six. The internal consistency/reliability for resilience in the present research, measured with McDonald’s omega (*ω*) was 0.74.

The questionnaire was carried out during the year 2024. The study was conducted in Romania via Google forms (Google LLC, Mountain View, CA, United States). Athletes practicing Olympic combat sports completed the BRS, including socio-demographic data, and regarding the highest sports performance obtained. The research is based on *ex post facto* design (Thomas and Nelson, 2001). We mention that 61 athletes (72.6%) completed the online questionnaire in the presence of the Romanian experimenters (athletes are, also, students at different physical education and sport faculties in Romania). These athletes were asked whether they know other athletes which meet the inclusion criteria in the study, and who practice Olympic combat sports (but not grappling Olympic combat sports, e.g., judo, wrestling). Therefore, another 31 answers were received after using the snowball sampling technique (the survey was anonymous). However, eight athletes were removed from the research because: two of them specified 1 year of competitive experience, three athletes mentioned that they were 17 years old at the time of testing, while three athletes had more than 38 years of age, which we considered to be a too long age gap in our sample. The results of the remained athletes (*N* = 84) were statistically processed. No incomplete questionnaires were received due to the mandatory fields when answering the questionnaire items.

### 2.3 Statistical analysis

IBM SPSS Statistics Version 27.0 (Armonk, NY, IBM Corp) and Jamovi (The Jamovi Project, 2024, Version 2.6) were used for the statistical analysis. In the case of analysis of variance Tukey *post-hoc* test was performed due to Levene’s test results for homogeneity of variants (*p* > 0.05). Variables do not deviate from normal distribution (Shapiro–Wilk test, *p* > 0.05), while Skewness and Kurtosis coefficients (in absolute value) < 2 (George and Mallery, 2010). The Goodman and Kruskal tau association was also used, following the recommendations of Argyrous (2005) – at least one variable being categorical, with Cramer’s V (effect size) range intervals (for 2 × 3 tables): 0.50 - strong association; 0.10 - weak association; 0.30 - moderate association (Nyberg et al., 2023). Not least, a binomial logistic regression was used, the effect size index (Nagelkerke R^2^) being interpreted as follows: 0.35 large effect size, 0.2 small, respectively 0.15 medium effect size (Cohen, 1992). The level of significance was set at *p* < 0.05 (for the null hypothesis to be rejected). The rationale for using binomial logistic regression is that the dependent variable/criterion (sports performance) is categorical and dichotomous (Christensen, 2005).

## 3 Results

Data were normally distributed, and the homogeneity condition was met (Levene’s test, *p* > 0.05), important assumptions for analysis of variance. We did not find sport-related differences in terms of psychological resilience (*F* = 1.66, *p* = 0.182, *M*_BOXING_ = 3.92, *M*_KARATE_ = 3.86, *M*_TAEKWONDO_ = 4.21, respectively *M*_FENCING_ = 4.02), Table 2 presents the descriptive statistics for sport.

**Table 2:** Descriptive statistics – type of sport.

Variable	Type of sport	*N*	Mean	SD	SE
Resilience	Boxing	24	3.92	0.445	0.090
	Karate	26	3.86	0.529	0.103
	Taekwondo	16	4.21	0.497	0.124
	Fencing	18	4.02	0.556	0.131

Second, we investigated if there are gender-related associations in terms of resilience, in combat sport athletes. Goodman and Kruskal tau association test was used, the results for resilience representing the dependent variable (Table 3).

**Table 3:** Goodman and Kruskal tau association - directional measures.

Association between variables		Value	Asymp. std. error	*p*
Goodman and Kruskal tau	Gender	0.211	0.078	< 0.01
	Resilience dependent	0.105	0.040	< 0.01
Crosstabulation Gender and psychological resilience				
	Resilience			Total
	Average results (between 3–3.69)	Slightly above average scores (3.70–4.30)	High scores^*^ (more than 4.30)	
Male athletes	13	21	27	61
Female athletes	15	7	1	23

*Smith et al. (2008).

Out of the 61 male combat sport athletes, 13 athletes (21.3%) registered average scores (between 3 and 3.69) in the case of psychological resilience, 21 (or 34.4%) obtained slightly above average values (between 3.7 and 4.3), while 27 athletes (44.2%) registered high scores (> 4.3). With respect to the 23 female combat sport athletes, 15 (65.2%) obtained average scores for resilience, 7 (30.4%) registered slightly above average values, and only one athlete registered a high score (see Table 3).

The Goodman and Kruskal tau association coefficient is 0.105, while the *p* < 0.01. A significant association was found between athletes’ gender and the results for psychological resilience. Cramer’s V coefficient is 0.46, highlighting a strong link between variables.

The existing differences between martial artists, according to sports performances, were, also, investigated using analysis of variance (*F* = 6.61, *p* = 0.002). Tukey *post-hoc* test was used (Table 4) and descriptive statistics for sports performance are presented in Table 5.

**Table 4:** Tukey *post-hoc* test – psychological resilience.

Sports performance		I	*N*	R/L
I	Mean difference	—	0.086	−0.355
	*p*-value	—	0.782	0.024
N	Mean difference		—	−0.441
	*p*-value		—	0.002
R/L	Mean difference			—
	*p*-value			—

I, international performances; N, national level results; R/L, regional/local results.

**Table 5:** Descriptive statistics – resilience level according to athletes’ sports performances level.

Variable	Performances	*N*	Mean	SD	SE
Resilience	I	26	3.89	0.438	0.085
	*N*	31	3.81	0.540	0.097
	R/L	27	4.25	0.447	0.086

I, international performances; N, national level results; R/L, regional/local results.

Data analysis revealed significant differences between athletes having national and/or international sports performances and athletes having regional and/or local sports results (Table 4, *p* = 0.02, respectively *p* < 0.01). Elite combat sport athletes registered a slightly above average level of resilience (*M* = 3.89, *SD* = 0.438), while athletes with lower results in competitions obtained the highest scores (*M* = 4.25, *SD* = 0.447, see Table 5).

In the next phase, knowing that psychological resilience is specific to combat sport athletes having international and national performances, the extent to which resilience predicts sports performances was examined (*n* = 84). A binomial logistic regression was performed (Tables 6, 7).

**Table 6:** Binomial logistic regression analysis – psychological resilience.

Psychological resilience	
Chi-square and *p*-value (Omnibus test)	11.913 and 0.001
R^2^_N_	0.185
Regional/Local sports results (predicted)	37% - Sensitivity
International and national sports performances (predicted)	87.7% - Specificity
Overall percentage (Predicted - Percentage correct)	71.4% - Accuracy
Chi-square and *p*-value (Hosmer and Lemeshow test)	4.57 and 0.712

R^2^_N_: effect size (Nagelkerke R square).

**Table 7:** Variables in the equation - binomial logistic regression (resilience).

Variable	B	S. E.	Wald	df	*p*	Exp(B)/odds ratio	95% C.I. for Exp(B)	
							Lower	Upper
Resilience constant	−1.71	0.537	10.121	1	0.001	0.181	0.063	0.519
	7.668	2.221	11.916	1	0.001	2139.029		

The Omnibus test checks the overall significance of the regression model - the model is significant (*p* < 0.01, Table 5). The *p*-value is 0.71 for the Hosmer and Lemeshow goodness of fit test, meaning that the prediction model is suitable in relation to the research data. The total percentage of correct classification is 71.4%. The effect size R^2^_N_ = 0.185, highlighting a moderate to strong link between psychological resilience and sports performance.

In the case of athletes practicing Olympic combat sports, the results for resilience represent an important predictor of sports performance. A slightly above average value (at least 3.7, but lower than 4.3) for psychological resilience is associated with an increased likelihood of international and/or national performances in athletes. On the other hand, the highest scores for resilience were linked to a lower probability of achieving outstanding sports results.

## 4 Discussion

The aim of our study was to investigate psychological resilience in Olympic combat sports, according to type of sport, gender and sports performance. Also, we wanted to verify if resilience predicts sports performance in martial artists.

Our findings showed that combat sport athletes registered slightly above average results for resilience (*Mode* = 3.83) compared to normative data, meanwhile previous studies using BRS emphasized a mid-level of resilience in martial arts athletes (Patenteu et al., 2024; Kuçuk Kiliç, 2020). With regards of the type of sport no differences, in terms of psychological resilience, were found between the Olympic combat sports investigated (boxing, karate, fencing and taekwondo), the null hypothesis being accepted. Bingöl and Bayansaldüz (2016) also found no significant link between the practiced sports discipline (boxing, taekwondo, Muay Thai, wrestling) and the level of psychological resilience. Moreover, Blanco-García et al. (2021) using the Brief Resilience Scale (BRS) suggested no significant variations among sports.

Considering the differences in resilience between genders, our findings showed a significant association between combat sport athletes’ gender and the BRS scoring, with male athletes obtaining higher scores than female athletes (the null hypothesis was rejected). Similarly, a meta-analysis conducted by Gök and Koğar (2021) on gender differences in psychological resilience found that men consistently scored higher on resilience measures than women across a wide range of studies. This further reinforces the idea that gender-specific resilience interventions may be necessary to address these disparities. Not least, Blanco-García et al. (2021) underlined that the results considering psychological resilience were higher in males than in females (judo, athletics, handball, basketball and volleyball practitioners were investigated).

Psychological resilience is essential in sports field, a stress-generating environment (Mellalieu et al., 2009). But, can we talk about a level of resilience which facilitates performance in Olympic combat sports? In the present study, elite athletes with international and/or national sports experience registered a slightly above average level of resilience (between 3.7 and 4.3), while athletes with lower caliber (see McKay et al., 2022) obtained the highest scores. The fact that a slightly above average level of resilience, generally (according to the norms) is linked with a higher sports performance, while the highest values in the case of resilience are associated with lower sports results may be surprising. However, psychological resilience has a complex nature (Southwick et al., 2014), is operating at multiple levels involving characteristics such as optimism, self-efficacy, increased self-esteem (Connor and Davidson, 2003; Bonanno, 2004), and a higher level of optimism and self-esteem may also disrupt sports performance. As Patenteu et al. (2024) argued (referring to martial artists), optimism and too high level of self-confidence “experienced too early in competition, can reduce athletes’ fighting capacity, increasing the risk of injury” (p. 8). Pânișoară (2024) underlined two essential aspects of psychological resilience: (a) adversity, life situations that take us out of our comfort zone, and (b) solving the situation which helps the individual to integrate his/her experience (physical and mental) and adapt to the context. Taking into consideration the mentioned aspects, we can better understand the findings of the current study. Therefore, in the event of a defeat (and athletes with lower results in competitions have, in martial arts, more defeats on their record than those with outstanding sporting performances), athletes step out of their comfort zone, needing to be mentally and physically resilient (even more so in the event of an injury) to continue training and hope for superior results. More defeats in combat sports increase the likelihood of injury, so athletes (who have suffered more defeats) are more often out of their comfort zone, and resilience is needed to keep going. Interestingly, Patenteu et al. (2024) found a positive link between martial arts athletes’ resilience and aggression (Foul/violent play and Go-ahead factors). As the authors mentioned - “athletes who are more resilient tend to attack, to go forward no matter what (specific aspects of the Go-ahead factor), and also, these athletes who have set their sights on not giving up regardless of the obstacles encountered are more prone to unethical actions in sport” (Foul play factor), they may break rules and manifest a higher probability of violent behaviors. It seems that during low-level competitions in judo (athletes without outstanding results), a higher frequency of injuries was reported (Frey et al., 2004) and the time loss was between 21 and 29 days (Green et al., 2007), while the time loss (after injuries) during the Summer Olympic Games (London, 2012) was only 1–7 days (Engebretsen et al., 2013). In these conditions, resilience is essential in the process of injury rehabilitation (Neils-Strunjas et al., 2017; Codonhato et al., 2018) and can support the restoring of self-confidence which is in one’s ability to successfully perform and enables injured athletes to move beyond a mere return to sport to a return to high performance (Conti et al., 2019). Athletes having a high level of psychological resilience may be “better able to deal with obstacles, setbacks, and failure that come with sports competition” (Predoiu et al., 2023). However, there are studies emphasizing no significant association between injury severity and combat sport athletes’ performances (Patenteu et al., 2023). Also, it is worth mentioning that martial artists from striking combat sports (e.g., boxing, karate, taekwondo) “are significantly more resilient and score significantly higher on Foul (violent) play,” compared to athletes from grappling combat sports (jiu-jitsu, judo), being more willing to win at any cost and, at the same time, recover more easily after a difficult condition in life or in sports career (see Patenteu et al., 2024). Pedro (2016) finding revealed that engagement in combat sports (wrestlers were examined) and resilience are positively correlated. Engagement supposes effort, duration toward actions, intensity, enthusiasm (Skinner and Pitzer, 2012), but too much engagement, intensity, effort, and even enthusiasm (aspects positively linked with resilience) may, sometimes, impair performance. One can also think to theoretical models that linked arousal and performance (Yerkes and Dodson, 1908) which suggest the existence of an optimal level of activation to achieve peak performance. More recent approaches, such as the Individual Zone of Optimal Functioning (IZOF) model (Hanin, 2000), emphasize that the “ideal” emotional state is highly individualized, with idiosyncratic responses. In fact, a too low level of arousal can lead to poor stimulation and slow responses. On the other hand, an excessively high level of arousal can cause anxiety and a decrease in concentration. The IZOF model suggests that each athlete has a unique zone in which they perform at their best developing specific competences (Bertollo et al., 2016). Not least, it seems that persons with high resiliency better tolerate risky circumstances (McCleskey and Gruda, 2021), and that resilience predicts higher participation (Wardlaw et al., 2018). But a risky decision/ situation in combat sports does not necessarily mean a successful technical execution, instead may lead to injury and premature withdrawal from the competition.

Dahiya and Gupta (2021) found a high positive correlation between resilience and mental skills, respectively self-motivation, and a low positive link between resilience and athletic ability, character and emotional stability. Researchers used a self-reported instrument to measure the investigated dimensions (e.g., athletic ability, mental skills, character).

The results of the present study are all the more surprising considering that Hosseini and Besharat (2010) suggested that resilience was positively associated with sport achievement, psychological wellbeing, and negatively related with psychological distress (in the study most athletes practiced volleyball, basketball, football, running and swimming). Meggs et al. (2016) reported, also, that resilience influences sports performance directly and indirectly “through appraisal (i. e., interpretation of the stressor to be facilitative and non-threatening).” However, a positive interpretation of a stressful event can happen regardless of the athletes’ performance level, perhaps even more in the case of athletes aspiring to top performances, who may face more difficulties related to the training process (material base, financial aspects), compared to athletes who have already confirmed at international level. Interestingly (in line with our findings) – Meggs et al. (2016) argued that athletes having higher levels of resilience (in swimming) registered poorer performance than swimmers with lower levels of resilience. As authors mentioned – “one possible explanation […] is that the influence of psychological resilience (specifically protective factors) becomes less prominent in times of extreme physiological stress/adversity” (p. 172).

Even if in some studies it seems there is a consensus regarding the positive relation between psychological resilience and sports performance, there are, however, other studies showing divergent results. For example, no significant differences considering resilience were found between high-level athletes from individual or team sports and non-athlete university students (Boghrabadi et al., 2015). More than that, using the Brief Resilience Scale (the same tool used in the current research), Blanco-García et al. (2021) emphasized no significant differences on the level of resilience according to athletes’ competitive level (in handball, volleyball, basketball, judo and athletics). One can observe, therefore, certain contradictions regarding the relationship between resilience and sports performance, and when it comes to the relevance of resilience in different sports disciplines the findings are even more divergent. Reche-García et al. (2020) revealed that combat sport athletes have significantly higher levels of psychological resilience than the team or individual sports athletes, while other studies underlined no correlations between sport modality and athletes’ resilience levels (Bingöl and Bayansaldüz, 2016; Chacón-Cuberos et al., 2016).

The current study highlights, also, that in the case of athletes practicing Olympic combat sports, the results for resilience represent an important predictor of sports performance. More exactly, a slightly above average value for psychological resilience (between 3.7 and 4.3) is associated with an increased likelihood of international and/or national performance. On the other hand, the highest scores for resilience were linked to a lower probability to achieve top results.

To shed more and more light on combat sport practitioners’ resilience and how athletes bounce back following adversity, a tailored and multidisciplinary approach is required. Den Hartigh et al. (2022) emphasized (referring to resilience in sports) the necessity “to detect warning signals in the psychological and physiological data,” in athletes, using, for example, algorithms, sensors and/or apps. The technological integration into psychological skill training can lead to optimal consistent performances (Siekańska et al., 2021).

### 4.1 Limitations and directions for future research

Although our findings addressed gaps in the literature, regarding the link between psychological resilience and combat sport athletes’ performances, the current research has some limitations. First, the study was carried out only in Romania and the results could be different in another country or setting. Moreover, the sample size is not very large, and further studies could separately address the characteristics of each sport branch (e.g., boxing, fencing, etc.), a specific age and, also, each weight class in taekwondo or boxing, or each weapon in fencing (sword, foil, sword). The sample is unbalanced in terms of gender. However, there is a relatively equal distribution of female athletes when sports performance-related differences were investigated.

Considering the research design (*ex post facto* design), longitudinal research should delve deeper into investigating the observed relationships between psychological resilience and sports performance or athletes Caliber, in combat sports. In addition, athletes’ history of injuries (injury severity) could be considered in future studies, as well as variables such as nutrition, recovery, athletes’ exercise capacity and sleep quality. BRS can be applied taking into consideration a specific moment, for example before a competition, or immediately after a competition. Not least, athletes from grappling combat sports (e.g., Greco-Roman wrestling, freestyle wrestling, judo) should be, also, investigated, Olympic combat sports involving throws and immobilization of opponent’s body.

## 5 Conclusion

The current study emphasized that a slightly above average level of psychological resilience is associated with an increased likelihood of international and/or national performances in Olympic combat sports. On the other hand, high scores for resilience (> 4.3) were linked to a decreased probability of achieving outstanding sports results. Additionally, it was found a significant association between combat sport athletes’ gender and the results for psychological resilience, with male athletes obtaining higher scores than female athletes. No significant differences between the Olympic combat sports investigated (boxing, karate, fencing and taekwondo) were highlighted, in terms of resilience. The findings offer a valuable window into understanding psychological resilience in combat sports.

## Data Availability

The raw data supporting the conclusions of this article will be made available by the authors, without undue reservation.
